# Switching from a High-Fat to a Regular Chow Diet Improves Obesity Progression in Mice

**DOI:** 10.3390/cimb47100791

**Published:** 2025-09-23

**Authors:** Yuying Wang, Fenglin Chen, Xiaozhong Wang, Shiwan Wang, Lei Ding

**Affiliations:** 1 Department of Gastrointestinal Surgery, Capital Medical University Affiliated Beijing Shijitan Hospital, Beijing 100038, China; 2 Department of Gastroenterology, Fujian Medical University Union Hospital, Fuzhou 350001, China

**Keywords:** obesity, comorbidities, high-fat diet, regular chow diet

## Abstract

The fast-paced lifestyle of modern people has changed their dietary structure and increased the prevalence of obesity, of which a high-fat diet is the main cause. Therefore, this study investigates whether reducing fat intake can improve obesity and physical health. We induced an obese model with a 60 kcal% fat diet (HFD) for 12 weeks, followed by an intervention with a 4.9 kcal% fat diet (regular chow diet, RD) for 20 weeks. We found that after 20 weeks of RD, various indicators were significantly reduced compared with the HFD group, including dietary intake (3.26 ± 0.38 g, *p* < 0.01), Lee index (385.24 ± 14.22, *p* < 0.0001), blood glucose (8.75 ± 2.44 mmol/L, *p* < 0.01), blood lipids (TC: 2.60 ± 0.63 mmol/L, *p* < 0.001; TG: 0.72 ± 0.08 mmol/L, *p* < 0.001; and LDL-C: 0.57 ± 0.30 mmol/L, *p* < 0.0001), and inflammatory status (IL-6: 32.70 ± 7.55 pg/mL, *p* < 0.05). In addition, increasing dietary intake also indirectly increased fiber intake, which could promote intestinal microbiota diversity. Changing the diet of obese mice from HFD to RD still maintained the abundance of the probiotics *Akkermansia*, *Parabacteroides*, *Alloprevotella*, and *Porphyromonadaceae*, among which fiber intake played an important role. Therefore, we found that only reducing dietary fat intake was effective for weight loss, and dietary fiber intake helped maintain a healthy intestinal microbiota balance.

## 1. Introduction

With the development of modern life, the global obesity problem is becoming increasingly serious, with the consequent development of metabolic diseases such as type 2 diabetes, non-alcoholic fatty liver disease (NAFLD), and coronary heart disease [1,2]. Obesity is an imbalance between calorie intake and energy expenditure, and excess intake promotes the expansion of adipose tissue [3]. While obesity is mostly induced by high-fat and high-energy diets [4], metabolic abnormalities caused by obesity may lead to hyperglycemia, insulin resistance (IR), oxidative stress, immune dysfunction, activation of chronic low-grade inflammatory chemokines, and macrophage infiltration [5]. Although dieting can help to produce weight loss in the short term, the weight will rebound as it is difficult to maintain. In addition, energy intake and dietary structure can affect obesity [6], among which high-fat diet (HFD) and large portion sizes are the main causes [7].

Obesity also activates the lipopolysaccharide/toll-like receptor 4 (LPS/TLR4) inflammatory pathway, leading to intestinal microbiota dysbiosis and increased intestinal barrier permeability [8,9]. Diet composition strongly influences intestinal microbiota: low-fiber, high-protein, and high-fat diets have been shown to promote inflammation and increase intestinal permeability [10]. Evidence from germ-free mice demonstrated that transplantation of feces from conventionally raised mice induced a 57% increase in body weight, highlighting the causal role of gut microbiota in weight regulation [11]. Moreover, dietary habits, lifestyle factors (exercise, alcohol consumption), probiotic or antibiotic use, and metabolic diseases can alter the abundance and diversity of intestinal microbiota [12,13]. Certain dietary interventions, such as polyphenols and fiber, can increase beneficial bacterial populations such as *Lactobacillus* and *Bifidobacterium*; short-chain fatty acids (SCFAs), as metabolites of dietary fiber fermentation, regulate colonic Treg cells and reduce enteritis and dysbiosis [14]. Natural compounds such as blueberry extracts [15], conjugated linoleic acid [16], tea catechins [17], and raspberry polyphenols [18] have also been reported to ameliorate obesity-induced metabolic and microbiota disorders.

Despite these advances, a critical gap remains: most previous studies have focused on the role of specific nutrients or bioactive compounds in regulating obesity and gut microbiota, whereas fewer investigations have evaluated the effects of modifying overall dietary structure, such as reducing high-fat intake while increasing fiber content, on obesity and its related metabolic disturbances. Addressing this gap may provide more practical and sustainable strategies for obesity management.

Therefore, the present study aims to investigate the effects of dietary structure modification—specifically, reducing HFD intake and increasing dietary fiber—on obesity, intestinal inflammation, and gut microbiota composition in obese mice.

## 2. Materials and Methods

### 2.1. Feeding of Mice

Thirty C57BL/6J mice (5 weeks old, male, 15 ± 0.7 g) were purchased from Beijing Huafukang Biotechnology Co., Ltd. (Beijing, China) and housed in the Experimental Animal Center of Fujian Medical University (Fuzhou, China). After 1 week of adaptation feeding, 30 mice were randomly divided into three groups: regular chow diet group (RD group, number (n) = 10) fed 4.9 kcal% fat diet for 32 weeks, HFD group (HFD group, n = 10) fed 60 kcal% fat diet for 32 weeks, and HFD-RD group (HFD-RD group, n = 10) fed 60 kcal% fat diet for 12 weeks and then changed to 4.9 kcal% fat diet for 20 weeks (Table 1). Since the incisors of mice are constantly growing, the hardness of the food will affect the length of the teeth, thus changing the dietary intake [19]. Therefore, teething sticks were placed in each cage to prevent the effect of softer HFD food on mouse feeding. Bedding was changed every 2–3 days, and the mice were observed for food and activity.

All mice were housed in an environment with a temperature of 22 ± 1 °C, a humidity of 55 ± 5%, and an automatic 12 h (h) light and dark cycle and were allowed to eat and drink freely. This study was approved by the Institutional Animal Care and Use Committee (IACUC) of Fujian Medical University and followed the Guide for the Care and Use of Laboratory Animals (approval number: FJMU IACUC 2020-0019).

### 2.2. Routine Measurements

Total food consumption for each cage was calculated by weighing food consumption on Tuesday, Thursday, and Sunday nights. Fasting blood glucose and body weight were measured every Monday after fasting overnight every Sunday. The obesity model is characterized by increased weight due to fat accumulation, but a short body length; so the Lee index is significantly increased. A ruler was used to measure the nose-to-anus body length; the Lee index was calculated at the end of week 32 as follows: body weight (g)^1/3^ × 10^3^/length (cm) [20].

For dietary intake, we added new food every Monday and calculated the remaining food in each cage on Sunday: “This week’s food − remaining food = this week’s dietary intake”; then the mean ± SD of each group (2 cages) was taken and divided by 5 to obtain the weekly intake of each mouse.

### 2.3. Serum Indicator Testing

At the end of week 32, fasting blood glucose was measured in the mice after overnight fasting, and tail blood specimens containing sodium citrate anticoagulant were collected to detect hemoglobin A1c (HbA1c). Blood was collected after removing the mice’s eyes, and then serum was separated to test total cholesterol (TC), triglycerides (TG), low-density lipoprotein cholesterol (LDL-C), and high-density lipoprotein cholesterol (HLD-C) levels [21]. Serum insulin (INS), glucagon-like peptide 1 (GLP-1), and inflammatory factors (interleukin 6 (IL-6), tumor necrosis factor-α (TNF-α), and interleukin 10 (IL-10)) were determined by enzyme-linked immunosorbent assay (ELISA) kits purchased from Shanghai Yuxuan Biotechnology Co., Ltd., Shanghai, China. [22]. Homeostasis Model Assessment of Insulin Resistance (HOMA-IR) = fasting serum glucose × fasting serum insulin/22.5 [23].

#### 2.3.1. Tissue Staining Experiments

After blood was collected from the eyeball, the abdomen of the mouse was opened quickly, and about 3–4 cm of proximal colon tissue was obtained. The tissue was rinsed with cold PBS, fixed with 4% paraformaldehyde, dehydrated with 30%, 50%, 70%, 80%, 90%, 95%, and 100% ethanol solutions, transparentized with xylene I and II, and embedded in paraffin for longitudinal sectioning. Paraffin sections were dewaxed with xylene I and II, rehydrated with 100%, 95%, 90%, 80%, and 70% alcohol solutions and distilled water, stained with the Alcian Blue & Nuclear Fast Red Staining Kit (Beyotime Biotechnolog, Shanghai, China), and sealed with neutral resin to observe the thickness of the colon mucus layer [24]. Liver tissue was fixed with 4% paraformaldehyde, embedded in optimal cutting temperature compound, and stained with Oil Red O to identify intracellular lipid droplets [25,26]. Histological differences were then quantified and evaluated using Image J 1.52software.

#### 2.3.2. Microbial Sequencing

By the end of week 32, we collected mouse feces into liquid nitrogen freeze and performed sequencing experiments as soon as possible. DNA was extracted from mouse feces and 16S rRNA macro-genomic bacterial colony sequencing analysis was performed using an Illumina Miseq™/Hiseq™ sequencer (Sangon Biotech, Inc., Shanghai, China) [27]. The amplification region was V3-V4 with the primers 341F: CCTACGGGNGGCWGCAG, 805R: GACTACHVGGGTATCTAATCC.

#### 2.3.3. Statistics

We used SPSS 25.0 for statistical analysis; data were expressed as mean ± standard (mean ± SD); *p* < 0.05 was considered statistically significant. One-way or multi-way analysis of variance was used for statistical analysis, and the F test was used for analysis of variance. GraphPad Prism 8.4 was used for graphing, and Image J was used to quantify histological differences.

## 3. Results

### 3.1. RD Improves Obesity Symptoms in Mice

**Figure 1:**
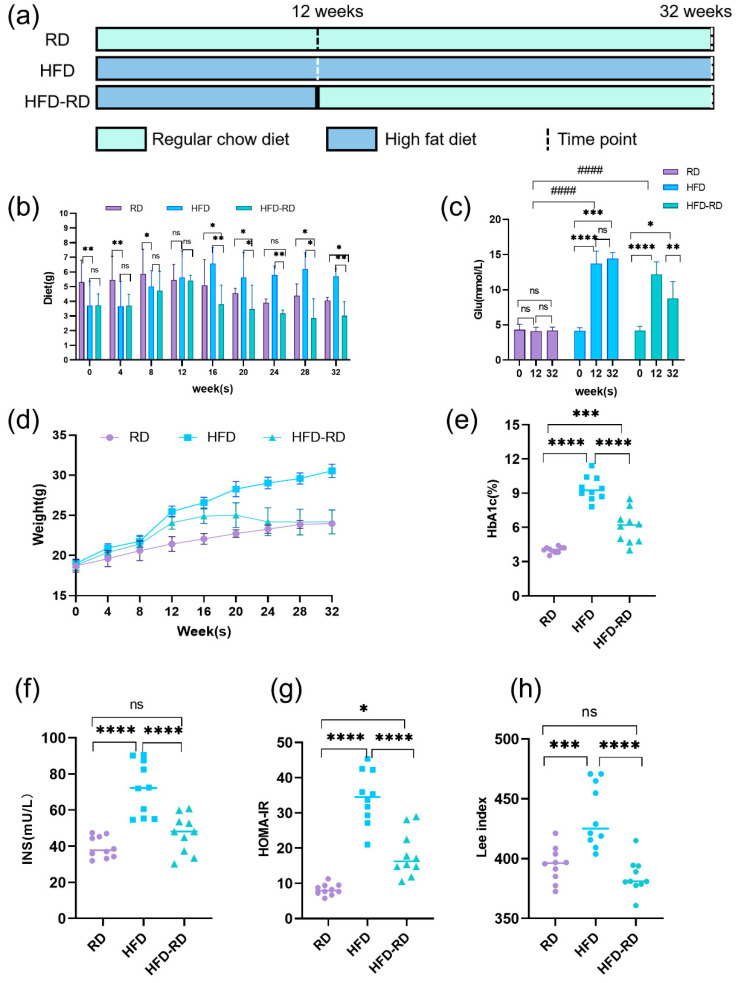
The obesogenic effect of dietary fat. (**a**) Experimental flow chart: including RD (regular chow diet), HFD (high-fat diet), and HFD-RD (high-fat–regular chow diet) groups. Regular chow diet: 4.9 kcal% fat diet, high-fat diet: 60 kcal% fat diet. There were 10 mice in each group. (**b**) Dietary intake was recorded every 4 weeks throughout the experiment to compare intake among the three groups of mice. (**c**) Fasting blood glucose levels were measured during the dietary changes to monitor metabolic changes in the mice. (**d**) Body weight of mice was recorded every 4 weeks and plotted as a line graph to assess growth trends and intergroup differences. (**e**) Hemoglobin A1c (HbA1c) levels in the three groups by the end of the experiment at week 32. (**f**) At the end of the 32nd week, serum insulin (INS) levels were tested. (**g**) At the end of the 32nd week, homeostasis Model Assessment of Insulin Resistance (HOMA-IR) = fasting serum glucose × fasting serum insulin/22.5. (**h**) Lee index at the end of 32nd week. ns: *p* > 0.05, *: *p* < 0.05, **: *p* < 0.01, ***: *p* < 0.001, ****: *p* < 0.0001, ####: *p* < 0.0001.

To investigate the effect of dietary fat content on mice, we fed mice in the HFD and HFD-RD groups 60 kcal% fat for 12 weeks. Compared to the RD group fed 4.9 kcal% fat for 12 weeks, the HFD and HFD-RD groups consumed less food at weeks 4 and 8 and consumed the same amount of dietary intake as the RD group at week 12 (Figure 1b). However, these two groups gained weight faster and had significantly higher fasting glucose (HFD group: 14.45 ± 0.84 mmol/L; HFD-RD groups: 8.75 ± 2.44 mmol/L) than the RD group (4.2 ± 0.49 mmol/L, *p* < 0.0001) (Figure 1c). Thereafter, the HFD-RD group underwent 20 weeks of RD, and food intake was maintained at a relatively stable low level (3.26 ± 0.38 g) after a period of rapid decline due to the change in dietary taste. The HFD group continued with 60 kcal% fat and the RD group continued with 4.9 kcal% fat until week 32, and their food intake was stabilized at a certain level after meeting growth needs (HFD group: 5.98 ± 0.40 g, RD group: 4.40 ± 0.47 g) (Figure 1a,b). In the HFD-RD group, by the end of week 32 compared to week 12, fasting blood glucose was significantly lower and at a relatively stable level (8.75 ± 2.44 mmol/L, *p* < 0.01) (Figure 1c). Meanwhile, by the end of week 32, blood glucose in the HFD-RD group (8.75 ± 2.44 mmol/L) was significantly lower than that in the HFD group (14.45 ± 0.84 mmol/L, *p* < 0.0001). In addition, HbA1c, which reflects long-term blood glucose levels, was significantly lower compared with the HFD group (6.05 ± 1.45, *p* < 0.0001) and was only slightly above the normal range of 4–6% (Figure 1e). At the end of week 32, the serological INS (*p* < 0.0001) and HOMA-IR levels (*p* < 0.0001) were also significantly lower in the HFD-RD group (INS: 46.90 ± 10.55 mU/L; HOMA-IR:18.17 ± 6.36) compared with the HFD group (INS: 72.04 ± 15.01 mU/L; HOMA-IR: 34.42 ± 7.57) (Figure 1f–g). The body weight was also significantly lower in the HFD-RD group and remained comparatively stable at low levels, and the Lee index was not statistically different from that of the RD group (385.24 ± 14.22, *p* > 0.05) and was significantly lower than that of the HFD group (HFD group: 435.94 ± 26.55, HFD-RD group: 385.24 ± 14.22, *p* < 0.0001) (Figure 1h). Based on this, we ended the experiment. And it was initially shown that changing the fat content in the diet had a positive effect on weight loss and lowering blood glucose.

### 3.2. RD Improves NAFLD in Obese Mice

**Figure 2:**
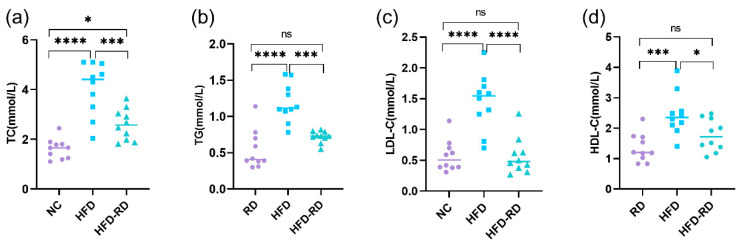
Effects of dietary fat on blood lipids and liver. (**a**) Serum total cholesterol (TC) levels were measured in mice at the end of the 32nd week to assess lipid metabolism. (**b**) Triglyceride (TG) concentrations in serum were evaluated at the 32nd week as an indicator of lipid accumulation. (**c**) Serum low-density lipoprotein cholesterol (LDL-C) levels were determined at the end of the 32nd week to reflect cardiovascular risk factors. (**d**) High-density lipoprotein cholesterol (HDL-C) levels in serum were assessed at the 32nd week to evaluate protective lipid profiles (RD: regular chow diet group, HFD: high-fat diet group, HFD-RD: high-fat–regular chow diet group; there were 10 mice in each group. ns: *p* > 0.05, *: *p* < 0.05, ***: *p* < 0.001, ****: *p* < 0.0001).

HFD also affects lipid storage and consumption, and excess lipids accumulate in the liver and adipose tissue and often combine with hyperglycemia to affect the health. At the end of week 32, blood was taken from behind the eyes, and serum was isolated to measure lipid concentrations. TC (2.60 ± 0.63 mmol/L, *p* < 0.001), TG (0.72 ± 0.08 mmol/L, *p* < 0.001), and LDL-C (0.57 ± 0.30 mmol/L, *p* < 0.0001) were significantly lower in the HFD-RD group compared with the HFD group (TC (4.05 ± 1.07 mmol/L), TG (1.19 ± 0.26 mmol/L), and LDL-C (1.45 ± 0.46 mmol/L)). In addition, HDL-C (1.77 ± 0.52 mmol/L, *p* < 0.05) was lower in the HFD-RD group compared with the HFD group (2.46 ± 0.70 mmol/L) and was not statistically different from the RD group (1.36 ± 0.46 mmol/L, *p* > 0.05) (Figure 2a–d).

### 3.3. Reducing Dietary Fat Improves Low-Inflammatory Status in Obese Mice

**Figure 3:**
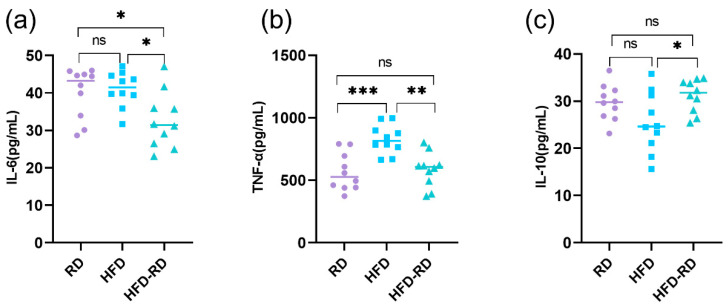
Serum inflammatory factors. (**a**) Serum levels of interleukin-6 (IL-6) were measured at the end of the 32nd week to evaluate systemic inflammatory responses. (**b**) Tumor necrosis factor-α (TNF-α) concentrations in serum were assessed at the 32-week endpoint as a marker of pro-inflammatory status. (**c**) The anti-inflammatory cytokine interleukin-10 (IL-10) was quantified in serum samples collected at the end of the 32nd week (RD: regular chow diet group, HFD: high-fat diet group, HFD-RD: high-fat–regular chow diet group; there were 10 mice in each group. ns: *p* > 0.05, *: *p* < 0.05, **: *p* < 0.01, ***: *p* < 0.001).

Obesity and its associated comorbidities are closely related to energy metabolic homeostasis and chronic inflammation [28]; so we measured serological inflammatory factors in mice after 20 weeks of RD intervention. The pro-inflammatory factors IL-6 (32.70 ± 7.55 pg/mL, *p* < 0.05) and TNF-α (584.65 ± 137.94 pg/mL, *p* < 0.01) were lower in the HFD-RD group compared to the HFD group (IL-6: 41.03 ± 4.71 pg/mL; TNF-α: 826.91 ± 116.37 pg/mL), and the levels of TNF-α were not statistically different between the HFD-RD and RD groups (RD group, IL-6: 40.04 ± 6.69 pg/mL; TNF-α: 564.96 ± 150.21 pg/mL, *p* > 0.05) (Figure 3a,b). The anti-inflammatory factor IL-10 was higher in the HFD-RD group (31.14 ± 3.47 pg/mL) than that in the HFD group (25.45 ± 36.39 pg/mL, *p* < 0.05) and was not statistically different from the RD group (29.73 ± 3.81 pg/mL, *p* > 0.05) (Figure 3c). In conclusion, after 20 weeks of RD intervention, serum inflammatory factor concentrations were significantly reduced in the HFD-RD group, and the inflammatory response of obese mice was significantly improved.

### 3.4. Effect of RD on the Intestinal Microbiota of Obese Mice

**Figure 4:**
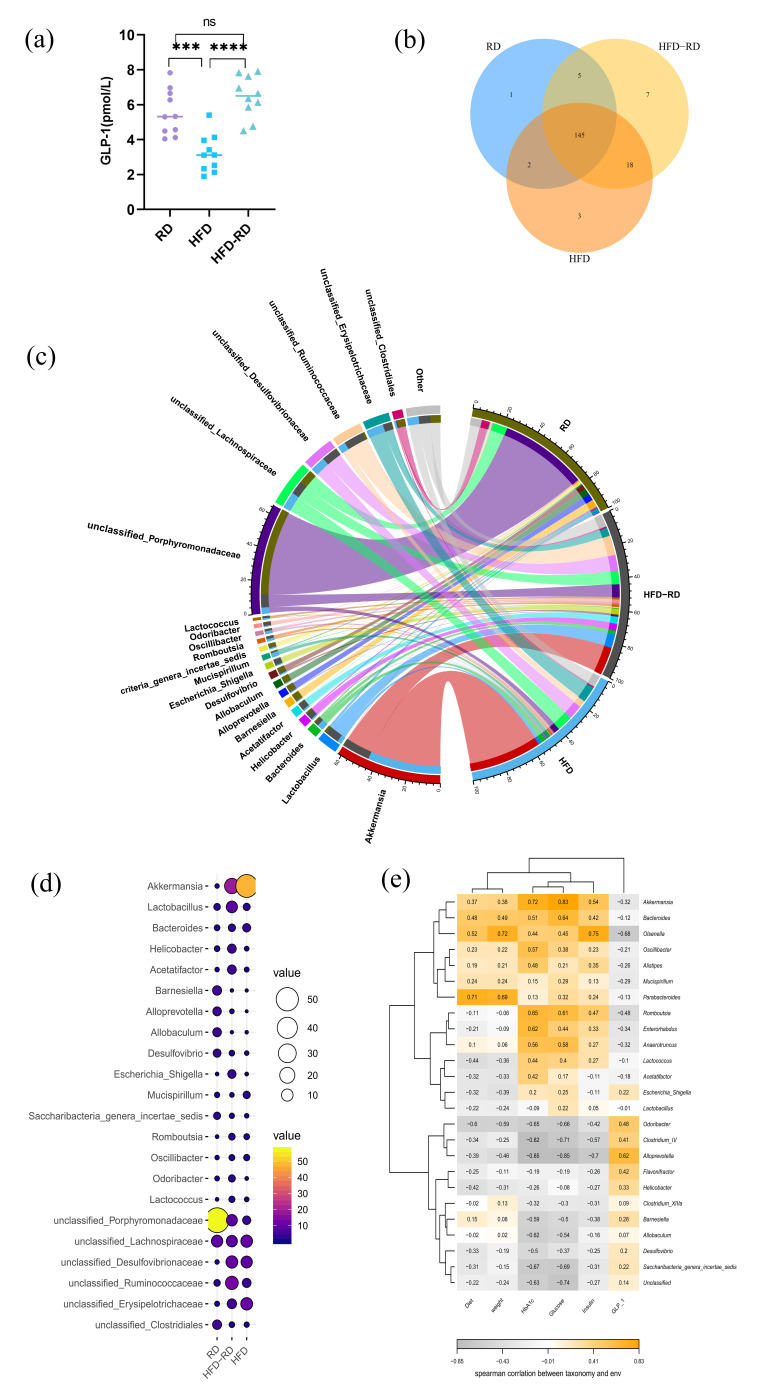
Intestinal microenvironment and microorganisms. (**a**) Serological glucagon-like peptide 1 (GLP-1) levels at the end of week 32. (**b**) Venn diagrams illustrating the shared and unique fecal microbial taxa among RD, HFD, and HFD-RD groups. (**c**) Circos plots depicting the distribution and relative abundance of fecal microbial communities in RD, HFD, and HFD-RD mice. (**d**) Bubble charts representing the differential abundance of fecal microbial taxa across RD, HFD, and HFD-RD groups. (**e**) Spearman correlation analysis of major microorganisms in mouse feces with obesity-related factors (RD: regular chow diet group, HFD: high-fat diet group, HFD-RD: high-fat–regular chow diet group; there were 10 mice in each group. ns: *p* > 0.05, ***: *p* < 0.001, ****: *p* < 0.0001).

We aimed to investigate whether the low-inflammatory state in obese mice is related to the intestinal microenvironment. Intestinal L-cell production of incretin GLP-1 usually has an ameliorating effect on obesity [29]. We examined GLP-1 in mouse serum and found that the HFD-RD group (6.46 ± 1.20 pmol/L) was significantly higher than the HFD group (3.21 ± 1.08 pmol/L, *p* < 0.0001) and was not statistically different from the RD group (5.56 ± 1.31 pmol/L, *p* > 0.05) (Figure 4a). To gain further insight into the changes in the intestinal microbiota of obese mice, we sequenced the feces of each group at the end of 32 weeks. After analysis, the highest diversity was found in the HFD-RD group, and the homogeneity of the distribution of the HFD-RD group was between the RD and HFD groups with a preference for the RD group (Figure 4b,c). Studies have shown that obesity and metabolic diseases are associated with changes in the relative abundance of bacterial species and specific genes in the gut microbiota [30]. Changes in dietary structure can affect the composition of microbiota, and the ratio of *Bacteria*/*Firmicutes* is negatively correlated with obesity and inflammation caused by irritable bowel [31,32]. And *Bacteroides* can improve obesity by producing SCFAs such as acetate and propionate [33,34]. The abundance of *Porphyromonadaceae* belonging to the phylum *Bacteroides* was highest in the RD group, and the abundance in the HFD-RD group also increased after RD treatment. Surprisingly, *Akkermansia* is a beneficial intestinal bacterium, and the increased fat intake in the diet contributes to the survival of *Akkermansia*. The highest abundance of *Akkermansia* was found in the HFD group, and this decreased slightly in the HFD-RD group after RD (Figure 4d). We further examined the correlation between intestinal microbiota and obesity, using Spearman correlation analysis to create a heat map of the major microorganisms and associated factors. The results showed that diet and body weight were positively correlated with *Parabacteroides* (*p* < 0.05); blood glucose and HbA1c were positively correlated with *Akkermansia* (*p* < 0.05); and *Alloprevotella* was significantly negatively correlated with blood glucose, HbA1c, and INS (*p* < 0.05), but significantly positively correlated with GLP-1 (*p* < 0.05) (Figure 4e).

### 3.5. Effects of RD on Histology in Obese Mice

**Figure 5:**
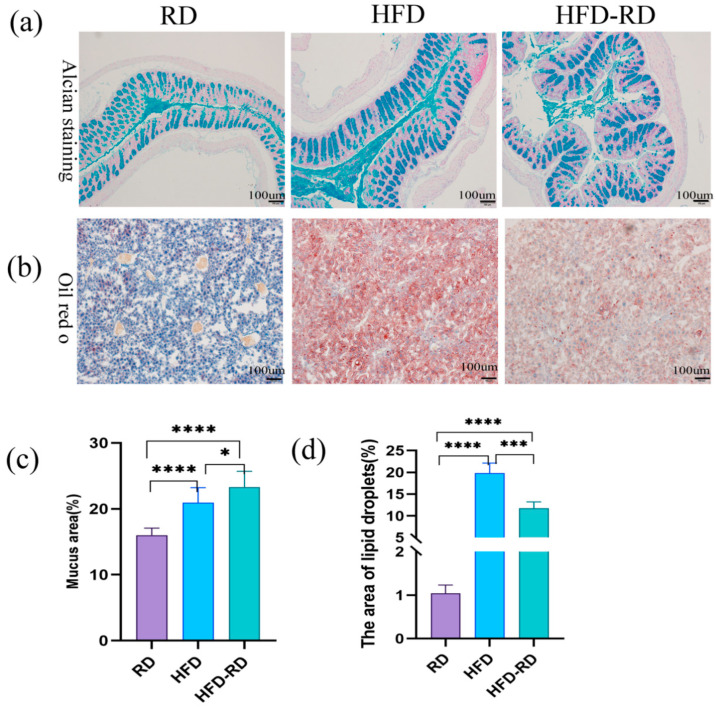
Colon and liver tissue staining. (**a**) Colonic Alcian staining was observed at 40× field of view. (**b**) Liver Oil Red O staining was observed in RD, HFD, and HFD-RD groups under 40× field of view. (**c**) Percentage of mucosal area in colonic Alcian staining. (**d**) The percentage of lipid droplet area after Oil Red O staining of the liver (RD: regular chow diet group, HFD: high-fat diet group, HFD-RD: high-fat–regular chow diet group; there were 10 mice in each group. *: *p* < 0.05, ***: *p* < 0.001, ****: *p* < 0.0001).

To further investigate the effect of RD on the histology of obese mice. Colonic tissue was taken for Alcian staining to observe the thickness and morphology of the colonic mucus layer. In comparison, the colon of the HFD-RD group had a richer blue mucus layer, normally arranged, and more numerous goblet cells compared to the HFD group (*p* < 0.05). Surprisingly, the HFD-RD group also had an abnormally abundant mucus layer with a relatively high number of goblet cells compared to the RD group (Figure 5a,c). To further investigate the effect of a reduced fat diet on the liver in obese mice, we performed liver Oil Red O staining to observe liver morphology. We found that the number of pseudolobules increased, and the hepatocyte structure was disturbed in the HFD group, while the number of pseudolobules was less in the HFD-RD group (*p* < 0.001), and there was a tendency to restore the normal liver structure. And the lipid droplets in hepatocytes of the HFD group were obvious, indicating the existence of a certain degree of NAFLD in HFD mice (Figure 5b,d). After 20 weeks of RD intervention, both the colonic mucus layer and liver tissue were significantly improved, as shown by an increase in the colonic mucus layer, a decrease in liver lipid droplets, and a decrease in the number of pseudolobules.

## 4. Discussion

The present study aimed to investigate whether reducing dietary fat content could improve obesity and its associated metabolic disorders in mice. Our findings demonstrate that switching obese mice from a high-fat diet (HFD, 60 kcal% fat) to a regular diet (RD, 4.9 kcal% fat) significantly reduced body weight and improved glucose tolerance [35,36], liver steatosis [37], and chronic low-grade inflammation [38]. Moreover, this dietary intervention promoted favorable alterations in gut microbiota composition, including increased abundance of beneficial bacteria such as *Akkermansia* and *Parabacteroides*. These results suggest that modification of dietary structure, rather than short-term caloric restriction alone, represents a practical strategy to alleviate obesity and its metabolic complications [39]. We selected RD (4.9 kcal% fat diet) for comparison with a 60 kcal% fat diet to study the effect of dietary fat content on mouse health. The aim was to investigate the improvement in obesity and its associated metabolic diseases in mice by reducing the fat content in the diet. We fed 6-week-old mice on a 60 kcal% fat diet for 12 weeks to construct an obesity model [40]. Food intake in mice is regulated by a model of homeostatic energy, and individuals usually dynamically adjust their energy intake according to their energy requirements to maintain their energy balance [41]. During the initial 12-week feeding period, the HFD-RD and HFD groups (60 kcal% fat diet) ate less than the RD group (4.9 kcal% fat diet) to meet survival needs. The HFD contains 60 kcal% fat; so eating less than the RD can lead to obesity. Subsequently, since this signal is processed by the brain largely outside awareness and is therefore quite resistant to conscious inhibitory control [42]; this increased food intake is referred to as a model of hedonic overdrive. The homeostatic and non-homeostatic/hedonic systems of the homeostatic energy regulation model interact, but the pleasure of eating fat can overturn the original energy balance [43], and this hedonic overdrive effect causes addictive behaviors of “liking” and “wanting” a high-fat diet in mice [44], driving individuals into a state of positive energy balance, leading to a state of obesity in the HFD-RD and HFD groups. The increased energy intake is associated with hypothalamic stimulation of the dopamine, opioid, and 5-hydroxytryptamine (5-HT) receptor systems [45]. All this suggests that the intake of higher levels of fat activates reward mechanisms that promote more food intake. Subsequently, as the fat content of the diet increased, mice had higher than normal blood glucose and lipid levels, IR, and NAFLD. The above experiments suggest that high-fat content in the diet can effectively induce obesity and related metabolic diseases.

Studies have shown that meal replacement and calorie restriction interventions for obese people can reduce both body weight and blood sugar [46]. If we simply reduce the fat content of the diet, will it facilitate weight loss in obese mice? Dietary energy density is positively associated with higher body mass index (BMI) values [47,48]. Therefore, we intervened with obese mice in the HFD-RD group with a 4.9 kcal% fat diet for up to 20 weeks. We replaced the sweet-tasting HFD with the normal-tasting RD, and the addiction established by the obese mice to HFD was forcibly blocked. Obese mice are in negative energy balance due to anorexia, and food intake is significantly reduced, while the high basal metabolic rate established by HFD is forced to consume already stored body fat due to the low intake of RD [49]. We observed a gradual weight loss in the HFD-RD group. By the end of the experiment, the Lee index was reduced by 10% compared to the non-intervention HFD group, and HbA1c, which responds to long-term blood glucose levels, was reduced by 35% and returned to normal HbA1c levels (6%). This suggests that the change in dietary fat content helped to improve body weight and glucose tolerance in mice in the presence of ad libitum activity [36]. Under the intervention of RD, mice were forced to adapt to RD and maintain low intake. The reduction in dietary energy intake reduced the metabolic burden on the liver. Firstly, serological lipid index examination showed that TC, TG, and LDL-C were significantly decreased. And HDL-C, which has antioxidant and anti-inflammatory effects, was significantly increased in the mice of the HFD-RD group. Secondly, histological examination showed that the liver in the HFD-RD group developed to a healthy state, and the lipid droplets of NAFLD were significantly reduced.

Chronic low-grade inflammation is an important feature of obesity [50], and overexposure to HFD leads to oxidative stress and inflammation [51]. Therefore, this study tested whether a 20-week RD dietary intervention could improve the low-grade inflammatory state in obese mice. Obesity-induced inflammation is thought to be the mechanism leading to IR and NAFLD [52], and HFD inhibits phosphorylation of AMPK-TBC1D1 signaling, increases protein levels of PPARγ, and ultimately promotes hepatic fat accumulation [53]. Obesity also recruits infiltrating macrophages, T lymphocytes, neutrophils, and dendritic cells (DCs) that produce associated inflammatory chemokines that exacerbate IR [54]. Serglycin is a proteoglycan that stimulates various immune cells in adipose tissue to express various pro-inflammatory factors such as IL-6 and TNF-α [55]. After RD intervention in obese mice, compared to the HFD group, serum concentration of pro-inflammatory factors IL-6 and TNF-α decreased, anti-inflammatory factor IL-10 increased, and the low-grade inflammatory state of obese mice was improved after RD.

The intestine is the first site of food digestion, and dietary composition directly influences the homeostatic regulation of intestinal mucosal immunity. Excessive fat intake alters the way the intestine metabolizes nutrients, causing intestinal inflammation, a risk that can be suppressed by dietary fiber [56]. Foods that cannot be digested by the intestine can be metabolized by intestinal probiotics to produce health-promoting polysaccharides and SCFAs. Depletion of various nutrients affects the structure of the microbiota and provides substrates for microbial metabolism. HFD causes dysbiosis, and the microbiota metabolite α-hydroxyisobutyric acid and LPS from Gram-negative bacteria co-activate the LPS/TLR4 signaling pathway, exacerbating the inflammatory response in obese mice [57]. Alcian staining of the colon after lowering dietary fat showed a thickened blue colonic mucus layer, an intact mucosal barrier, and reduced infiltration of inflammatory cells around the intestinal lumen in the HFD-RD group. Also, serological inflammatory factors were reduced in the HFD-RD group. Moreover, the gut–liver axis plays a prominent role in the pathological network of HFD-induced metabolic disorders [58], and the intervention of RD mediated the benign development of the liver in obese mice.

Microbiota can produce small molecules that are absorbed by the host and influence many important physiological processes, and changes in diet composition can affect the abundance and diversity of the gut microbiota. We collected feces from mice at the end of 32 weeks to analyze their microbes in relation to obesity and diet composition. Differences in the intake of specific nutrients could separate extensive changes in energy expenditure, gut microbiota, and metabolites from obesity. The HFD group had a high intake due to the long duration, along with increased fiber intake in the HFD, which would increase the diversity of the gut microbiota and also lead to increased energy metabolism [59]. This explains the rich microbiota diversity in the HFD group. The HFD-RD group constructed a rich microbiota diversity when induced as an obesity model and maintained the intake of fiber in the diet when changed to RD, and therefore was able to maintain high microbiota diversity.

*Bacteroides* has the ability to metabolize glutamate, which is a major component of MSG in daily life, and previous health nutrition investigations have shown that glutamate intake has the potential to promote the development of overweight [60,61]. Spearman correlation analysis showed that *Alloprevotella* had a significant positive correlation with GLP-1 and had a significant negative correlation with obesity-related factors. Forced feeding of *Bacteroides thetaiotaomicron* can reduce plasma glutamate and weaken diet-induced obesity [62]. In our study, the highest abundance of *Porphyromonadaceae*, which is a member of the *Bacillus* phylum and contributes to the reduction in visceral fat mass, was observed in the RD group, and increased in the HFD-RD group after RD intervention. It acts as an obesity regulator by producing SCFAs to increase lipolysis and fatty acid oxidation processes in adipocytes, thus reducing visceral fat accumulation and body weight [63]. Dietary fiber is an indispensable source of energy to maintain intestinal microbiota homeostasis and barrier integrity [64], and high fiber intake has a promotive effect on the growth of *Akkermansia* [65]. *Akkermansia muciniphila*, a mucin-degrading bacterium present in the mucus layer, stimulates the mucosal microbial network to improve intestinal barrier function by restoring mucus layer thickness and regulating tight junctions to reduce intestinal permeability in mice [66]. The abundance of *Akkermansia* in the HFD-RD and HFD groups was responsible for the thickening of their colonic mucus layer, which to some extent reduced the obesity-induced hypo-inflammatory state. Here, we confirm that high fiber intake, even when supplemented with high fat intake, promotes the growth of *Akkermansia*, and the high abundance of *Akkermansia* is maintained when the dietary structure is changed but a certain dietary fiber intake continues to be maintained. Therefore, weight loss may be more beneficial if the diet structure is changed to reduce fat intake while additionally increasing fiber intake [67]. Furthermore, the abundance of *Akkermansia* is inversely correlated with body weight [68], and its anti-NAFLD activity is associated with lipid oxidation and improves with increased levels of L-aspartate transported from the intestine in the liver [69]. This may also be the reason why HFD-induced T2D mice are generally less obese and pathogenic than STZ-induced T2D mouse models [40]. *Parabacteroides* have a wide range of bile acid conversion functions and are able to hydrolyze a variety of conjugated bile acids while converting them to a variety of secondary bile acids, which is positive for improving lipid metabolism disorders and maintaining the intestinal barrier integrity [70]. Parabacteroides is associated with weight status and eating behavior [71]. The relative abundance of Parabacteroides is positively correlated with the glycan structure containing terminal fucose. Dietary fiber can affect the O-glycosylation of colonic mucin, resulting in dietary fiber and SCFA metabolism affecting the abundance of Parabacteroides through mucosal glycans [72]. Therefore, *Parabacteroides distasonis* was significantly reduced during fasting or calorie-restricted diets. After the restoration of RD or HFD, the abundance of *Parabacteroides distasonis* increased with increased intake of dietary fiber [70]. In this study, *Parabacteroides* were positively correlated with diet (dietary fiber intake). However, as diet increases, body weight also increases; so *Parabacteroides* were positively correlated with body weight. *Alloprevotella* is a fiber-digesting bacterium in the colon, and fermenting fiber enhances SCFA production [73]. Fiber activates the liver phosphoinositide 3-kinase/protein kinase B/Forkhead box O1 (PI3K/AKT/FoxO1) and G protein-coupled receptor43/phospho-adenosine monophosphate activated protein kinase (GPR43/AMPK) signaling pathways and modulated the gut microbiota-SCFAs-GPR43/GLP-1 signaling axis, increasing GLP-1 level [74].

Taken together, our findings indicate that reducing dietary fat intake while maintaining sufficient dietary fiber not only alleviates obesity and related metabolic disorders but also promotes a beneficial gut microbiota profile, highlighting the crucial role of dietary structure in metabolic health. However, as this study was conducted in mouse models, further clinical research is needed to confirm the translational relevance in humans. In addition, while we mainly focused on dietary fat reduction, other nutritional factors may also play important roles in obesity management. Future studies combining dietary interventions with mechanistic validation and clinical trials will help to strengthen and extend our findings.

## 5. Conclusions

When developing a diet for obese individuals, a simple reduction in fat intake is easier to implement than an elaborate weight loss menu. A high-fat, high-energy diet can induce obesity in mice while causing hyperglycemia, hyperlipidemia, and damage to tissues such as the liver and colon. Replacing HFD with RD, reducing the fat content of the diet components while maintaining the fiber content of the diet can have the effect of reducing body weight, lowering blood glucose and lipids. In this study, only the fat content of the diet was changed. This alteration offers a new opportunity for weight loss in obese mice, as simply reducing dietary fat intake can effectively improve body weight and associated metabolic comorbidities in obese mice. Notably, if dietary fiber intake is increased along with reduced dietary fat intake, it will help the growth of intestinal probiotics and maintain the weight loss effect.

## Figures and Tables

**Table 1:** Mouse food composition.

Composition	High-Fat Diet (HFD), Grams	Regular Chow Diet (RD), Grams
Protein	203.00	180.00
Carbohydrate	197.80	176.60
Fiber	50.00	50.00
Fat	270.00	40.00
Mineral	50.00	25.22
Vitamin	3.00	1.34312
Dye	0.05	/
Total calories	60 kcal% fat	4.9 kcal% fat

Note: Feed content per kilogram. HFD, high-fat diet: D12492, 60 kcal% fat, Research Diets, lnc. (New Brunswick, NJ, USA); RD, regular chow diet: 4.9 kcal% fat (Fuzhou, China).

## Data Availability

The data that support the findings of this study are available in the methods of this article. Further details can be supplied from the corresponding author if required.
